# Hybrid Path Generation Method for Multi-Axis Laser Metal Deposition of Overhanging Thin-Walled Structures

**DOI:** 10.3390/mi15060704

**Published:** 2024-05-26

**Authors:** Han Liu, Fei Xing

**Affiliations:** 1 School of Mechanical Engineering, Shenyang University of Technology, Shenyang 110870, China; liuh@smail.sut.edu.cn; 2 Nanjing Zhongke Raycham Laser Technology Co., Ltd., Nanjing 210038, China

**Keywords:** additive manufacturing, computer-aided manufacturing, laser metal deposition, path planning, thin-walled structures

## Abstract

Additive manufacturing has advantages over other traditional manufacturing technologies for the fabrication of complex thin-walled parts. Previous correlation path strategies, when applied to laser metal deposition processes, suffer from contour deposition transboundary and surface “scar” type overstacking. Therefore, this paper proposes a hybrid path generation method for the laser metal deposition process. First, the topological logic of the STL model of the part is restored to reduce redundant calculations at the stage of obtaining the layered contour. Then, the path points are planned on the basis of the offset contours in a helical upward trend to form a globally continuous composite path in space considering the melt channel width. Finally, vectors that adaptively fit to the model surface are generated for the path points as tool orientations and they are optimized by smoothing the rotation angles. The results of experiments conducted on a multi-axis machine equipped with a laser metal deposition module show that the path generated by the proposed method is not only capable of thin-walled structures with overhanging and curved surface features but also improves the surface imperfections of the part due to sudden changes in the angle of rotation while ensuring the boundary dimensions.

## 1. Introduction 

With the rapid development of the equipment manufacturing industry, thin-walled structures are increasingly used in equipment and engineering in various fields. This type of structure has the advantages of lightweight and compact size, which is conducive to improving the efficiency of the whole machine and the working performance of the system [1]. When manufacturing complex thin-walled structures with overhangs and curved features, additive manufacturing (AM) technology offers shorter cycle times, higher degrees of freedom, and lower costs than traditional manufacturing techniques [2]. The technology is based on the principle of discrete stacking, which allows the machine to build up material layer by layer according to a planned path to form the part. In the additive manufacturing process, different materials such as metals, ceramics, and polymers are used in order to fulfill different production requirements. Laser metal deposition (LMD) technology is an important branch of metal additive manufacturing and is used in high-end manufacturing for the production of high-performance and rare metal components. It is based on the principle of using a high-energy laser to form a molten pool at a specified location, which absorbs and melts the metal powder, causing the material to go through the process of high-temperature liquid to cooled solid in a short time [3]. As a result, the traces left by the laser scanning along a defined path can be accumulated to form a part that matches the CAD model. However, if the pre-planned path is not reasonable, it can affect the quality of the final molded part or even lead to the failure of the task. Therefore, computer-generated path data are very important for additive manufacturing to produce parts [4].

At this stage, there have been many studies on generating paths, which can be categorized into 3-axis and multi-axis paths based on the number of axes of the equipment used [5]. For 3-axis paths, only two stages of slicing and path planning are required to obtain the coordinate points representing the deposition positions. Moreover, by moving the deposition head layer by layer in the X-Y plane, only 2.5-axis linkage is performed in the practical application of additive manufacturing. However, for multi-axis paths, the deposition head needs to be linked by multiple axes to adapt to the curvature changes in the part model surface, so it cannot be uniformly used only with a fixed *Z*-axis but rather with additional matching tool orientation for each coordinate point. As a result, slicing and path planning no longer remain independent of each other, but rather are strongly coupled.

### 1.1. Slicing for Additive Manufacturing

Any AM process has a similar process planning flow, where the model is first sliced based on layer-by-layer stacking. There are many slicing algorithms used for layering, and since the proposed composite path is applied on a planar substrate, planar slicing is recalled. Initially, some slicing methods with uniform layer thicknesses were the first to appear [6,7,8], aiming not only to obtain layered contours but also to improve the efficiency of computational results. Typically, they optimize the computation in two phases: intersection solving and contour building. Later, adaptive thickness slicing methods [9,10,11] appeared in order to improve the step effect due to discretization in regions with surface features [12]. However, the effect of adaptive slicing is limited, and changing the thickness affects the subsequent process. Wasserfall [13] also argued that such algorithms do not consider smooth transitions between layer thicknesses and are cumbersome in post-processing, which has led to the fact that adaptive slicing has not been widely used. Therefore, for the LMD process, uniform planar slicing is more suitable for obtaining the layered contours of metal parts.

### 1.2. Multi-Axis Path Planning

Regarding path planning, the common zigzag path and contour path-planning methods are prone to defects and sharp turns and are discontinuous [14]. Therefore, composite paths appear, which are algorithms that combine other strategies based on the characteristics of the slice layer. The contour method is usually chosen to fill the contour boundary region to ensure the geometric reduction in the boundary contour, and then the zigzag path is chosen to fill the solid part between the inner and outer contour to ensure the filling densities of the sliced layer, which is widely used in additive manufacturing technology [15].

Yigit et al. [16] concluded that paths formed on planar slices cause defects at the seam joints of the deposited layers, so they proposed a helical path method and verified the improvement on thin-walled parts with small overhangs using FDM technology. Zhao et al. [17] proposed a novel helical infill path to print parts on planar surfaces using a four-DOF rotary device. Bhatt et al. [18] also applied the helical path to a five-axis printer to meet the needs of unsupported manufacturing processes with larger overhang angles.

### 1.3. Conclusions of the Literature Review

For the LMD process, the helical path planning method is not perfect at this stage, and some details need to be paid attention to for path generation. For example, the path should be offset according to the melt channel width parameter, the tool should be oriented to the vertical deposition position, and the motion of the rotating equipment should be smooth. Failure to take these factors into account can affect the forming of parts that do not meet expectations in the process leading to the actual application of the path. Therefore, in order to solve the above-mentioned problems, the method in this paper is inspired by the composite method, which combines contouring and helical strategies to propose a composite path generation method. Depositing according to this path not only maintains the continuity of the path but also ensures a geometric reduction in the contour. In addition to this, the multi-axis tool orientation is rationally planned for two different states, vector and rotation angle combination, in order to fabricate an unsupported overhanging thin-walled structure with better surface quality.

The rest of the paper is organized as follows. In Section 2, the methodology for generating hybrid paths is described in detail from the various stages of slicing, path planning, and tool orientation generation. Section 3 deposits parts to verify the effectiveness of the method and the results are analyzed and discussed. Section 4 provides conclusions of the proposed method.

## 2. Methodology

**Figure 1:**
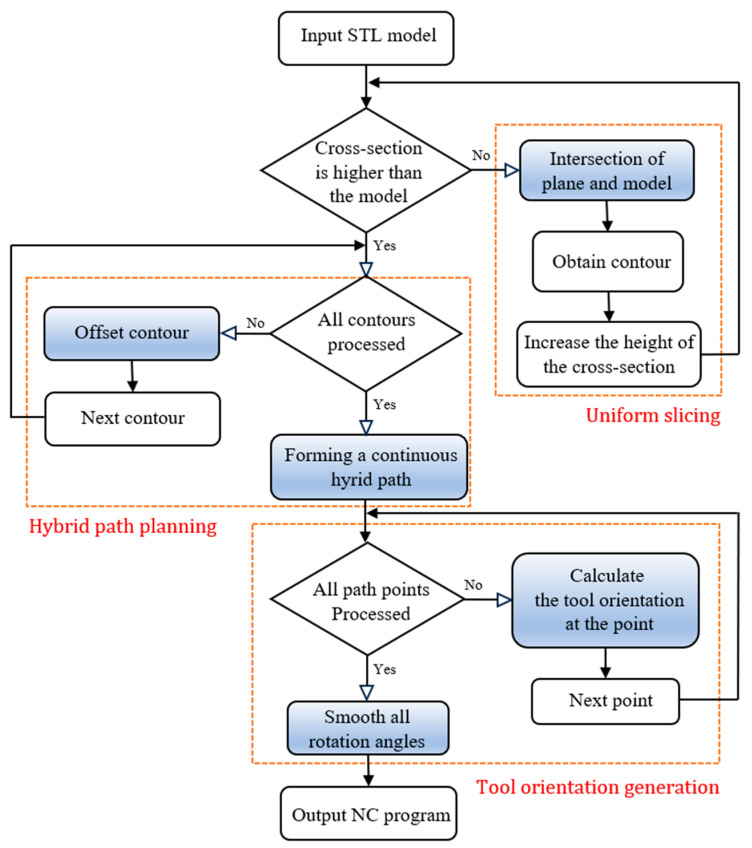
Workflow for the proposed path generation method. The boxes mark the three main modules, and the blue nodes in each module are their focuses.

The purpose of the method in this paper is to provide a multi-axis hybrid path for the LMD process to fabricate metal parts with unsupported overhanging thin-walled structures and to maintain a good appearance and surface quality. As shown in Figure 1, the method is divided into three main stages: uniform planar slicing, hybrid path planning, and multi-axis tool orientation generation. 

### 2.1. Uniform Planar Slicing

**Figure 2:**
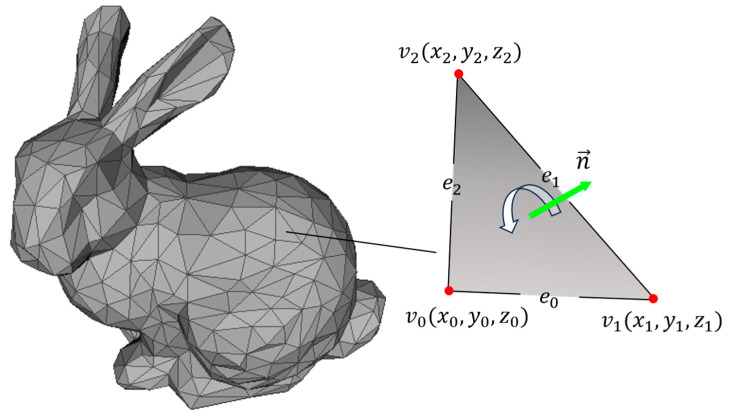
Model in STL format. As an example, the triangular mesh of Bonnie Bunny gives the main properties of one of the triangular facets.

Planar slicing is used to achieve layering of the part model, and a common delivery format in additive manufacturing is stereolithography (STL) [19]. The STL file contains an unordered collection of triangular facets that are used to fit the surface of the part model. Each triangular facet slice has three vertex coordinates ordered in a counterclockwise direction with a normal vector, as shown in Figure 2. The principle of uniform plane slicing is to use a set of planes with fixed intervals to perform intersection calculations with the STL model to achieve the effect of layering the part model. Therefore, the input of the slicing algorithm is a set of unordered lists of triangular facets, while the output is several ordered sets of intersections.

**Figure 3:**
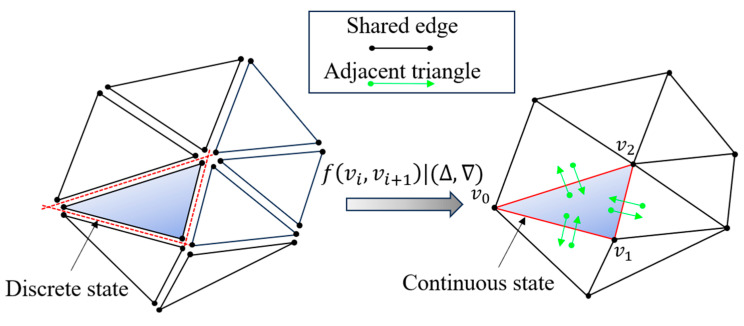
Restoration of the topological relationship. Reading the STL format yields a discrete set of triangular facets, which are transformed into a continuous state by constructing a dictionary.

First, a set of planes is obtained based on the highest vertex of the model and a given layer thickness. From the spatial location relationship, it is known that each plane is associated with only some of the triangular facets in the triangle mesh. Therefore, the list of triangular facets is grouped according to the heights of the planes, and only the triangular facets crossing the current plane are processed at a time. This can avoid a lot of invalid intersections to reduce redundant computations. This is iterated through each triangular facet grouping to restore their topological relationships. A dictionary is used to store the relevant information, and each of its entries contains the ordinal number of an edge and two triangular facets that share this edge. The ordinal numbers regarding the edges are obtained according to Equation (1).

(1) $$f\left(v_{i},v_{i+1}\right)=\left(v_{i}+v_{i+1}+2\right)\times \pi +\left(\left(v_{i}+1\right)\times \left(v_{i+1}+1\right)\right)$$

where $v_{i}$ and $v_{i+1}$ are the two endpoints of an edge. The effect after constructing the dictionary is shown in Figure 3 where the original triangular facets are all in a discrete state with each other, and their topological relationship is restored by the dictionary, thus changing to a continuous state. 

Then, a search is performed of edges that have intersections with the current plane according to a dictionary storing topological relations. In this case, the triangular facets in the dictionary are all in an intersectional relationship with the plane. The first edge is the longest edge of the first triangular facet in the dictionary, which consists of the lowest vertex and the highest vertex of the triangular facet. This type of edge must intersect the current plane, so it is recorded in the list of intersecting edges. In order to find the second intersecting edge of this triangular facet, it is only necessary to determine whether the height of the middle vertex is above or below the plane. If the middle vertex of the triangular facet is above the plane, then the intersecting edge is made up of the middle point and the highest point; otherwise, it is made up of the middle vertex and the lowest vertex. 

Only two edges of a triangular facet crossing a plane can intersect that plane, so the end of processing all but the first edge means that the current triangular facet has been used. Therefore, the second edge of the triangular facet we just processed is used as the keyword to search for another triangular facet in the dictionary that shares it, and the cycle of “edge-triangular-edge” searching continues by repeating the previous operation. The cycle continues until the first triangular facet is traversed again; thus, all intersecting edges of the current layer plane are obtained in an orderly sequence. 

**Figure 4:**
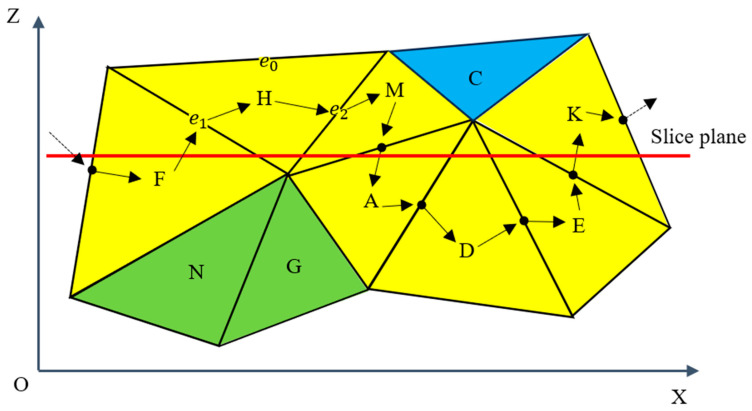
Determination of the edges that intersect the plane in an ordered manner. We find all edges of the yellow triangular facets that intersect the plane by traversing the dictionary in the current loop.

As shown in Figure 4, the yellow triangle facets are elements within the group, while the green and blue triangle facets belong to other groups and will not be processed in the loop of the current plane. After selecting any triangle facet H as a seed, we store the two edges $e_{1}$ and $e_{2}$ that it intersects with the plane and then finds another triangle facet M sharing $e_{2}$ according to the dictionary and judge and store its second intersecting edge. The cycle continues until it returns to triangle facet F to end the cycle, at which point all edges intersecting the current plane have been stored in order. 

Finally, to calculate the intersection of a line and a plane, the fact that the point of intersection is on both the line and the plane should be taken into account. Therefore, moving the reference point of the origin on the line a distance along that line is the point of intersection. Given the distance D from the plane to the origin O and its normal vector $N_{p}$, as well as the direction of the line $N_{l}$, we can find the distance t according to Equation (2).

(2) $$t=\left|\left(\left(O+O\cdot N_{l}\right)\cdot N_{p}\right)-D\right|\times {(N}_{p}\cdot N_{l})$$

**Figure 5:**
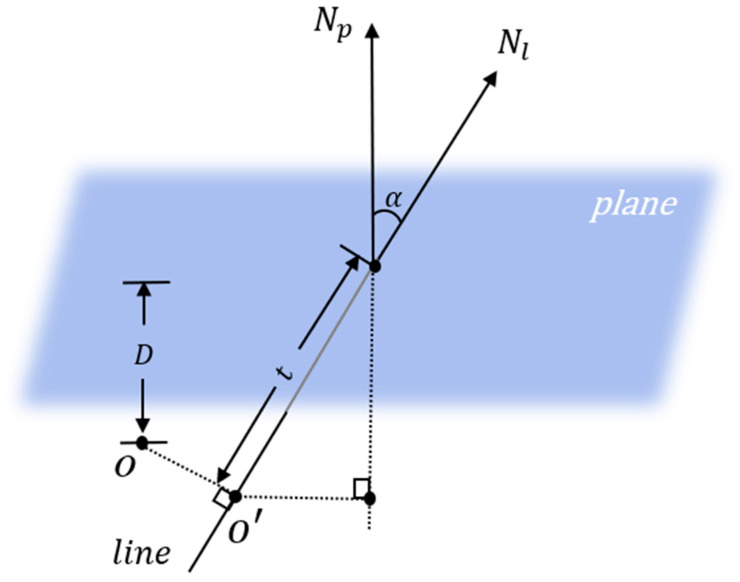
Plane and edge-seeking intersection calculations. We reduce the problem in dimension and use projections and geometric relationships to obtain intersections.

Its principle is shown in Figure 5, where reference point A is obtained by projecting the origin O onto $N_{l}$, and cos⁡α is obtained by the dot product of $N_{p}$ and $N_{l}$. The distance from point A to the plane multiplied by cos⁡α is able to obtain t.

### 2.2. Hybrid Path Planning

**Figure 6:**
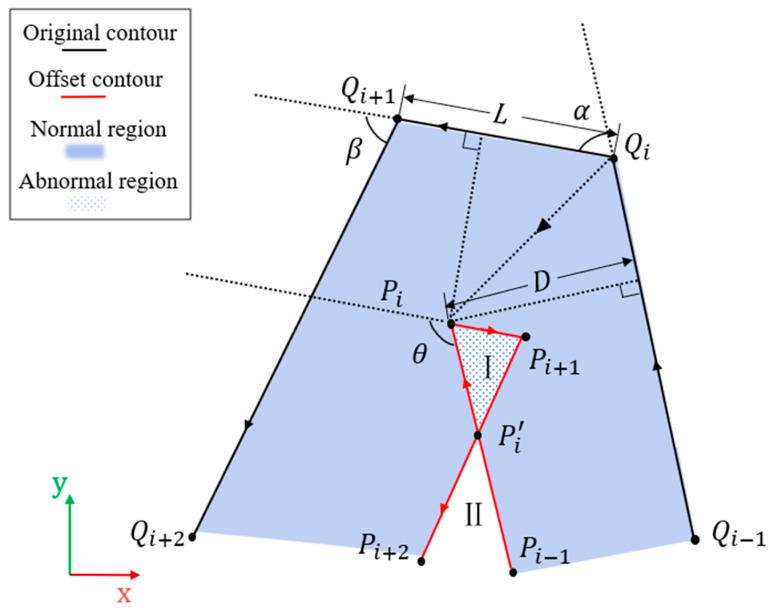
Contour offset. Detects and processes abnormal regions that appear to be self-intersecting, and preserves normal regions.

Before realizing the helical connection, the contour obtained by slicing needs to be offset inward. At this stage, there are many methods for offsetting polygons in the plane, but few of them can handle the self-intersection problem more stably and accurately. Because the methods in this paper usually do not require more complicated filling rules, we only focus on the case of local intersections here. As shown in Figure 6, the points on the original contour are offset and then the self-intersecting case is handled to form the new contour.

The coordinates of points $Q_{i-1}$, $Q_{i}$, and $Q_{i+1}$ are known as $\left(x_{i-1},y_{i-1}\right)$, $\left(x_{i},y_{i}\right)$, and $\left(x_{i+1},y_{i+1}\right)$ and the offset distance is d. The angle of θ is obtained according to Equation (3), and then the new vertices $P_{i}$ after offset are calculated according to Equation (4).

(3) $$\cos\theta =\frac{\vec{Q_{i}Q_{i+1}}\cdot \vec{Q_{i-1}Q_{i}}}{\left\|\vec{Q_{i}Q_{i+1}}\right\|\left\|\vec{Q_{i-1}Q_{i}}\right\|}$$

(4) $$P_{i}=Q_{i}+\frac{\left(\vec{Q_{i}Q_{i+1}}+\vec{Q_{i}Q_{i-1}}\right)}{2}\times \frac{d}{\sin\frac{\theta }{2}}$$

We repeat the above to obtain the remaining new points $P_{i-1}$, $P_{i+1}$ and $P_{i+2}$. Then, according to Equation (3) in the same way, we know angle α between $Q_{i-1}Q_{i}$ and $Q_{i}Q_{i+1}$ angle β between $Q_{i}Q_{i+1}$ and $Q_{i+1}Q_{i+2}$. The points $Q_{i}$ and $Q_{i+1}$ form a straight line L. According to the Pythagoreanrean theorem, we can obtain the distances $d_{i}$ and $d_{i+1}$ of the movement of $Q_{i}$ and $Q_{i+1}$ on L, respectively. If their sum is greater than the length of L, it indicates that the region where the new vertex is located has an anomaly. We then calculate the intersection $p_{i}^{\prime }$ of $P_{i-1}P_{i}$ and $P_{i+1}P_{i+2}$ in place of the anomalous line segment, and store only $P_{i-1}$, $P_{i}^{\prime }$, and $P_{i+2}$ in the list. When representing the two ring regions I and II formed, only the valid region II is retained.

**Figure 7:**
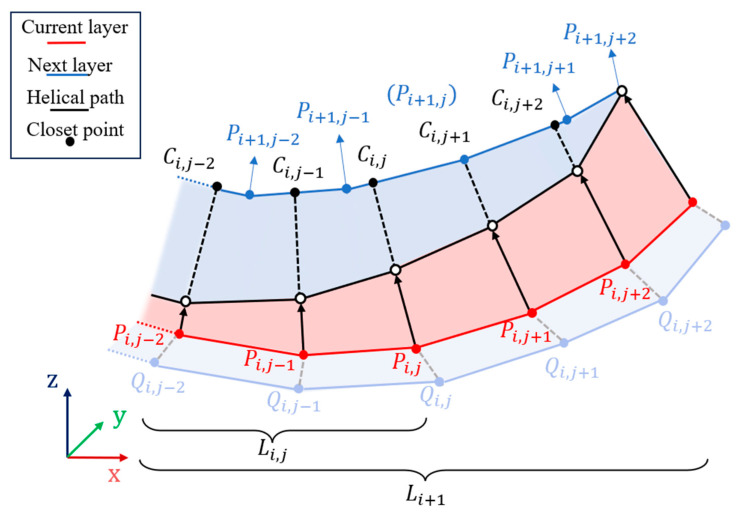
Generate composite paths. A continuous path is formed by connecting the contours of each layer that undergoes the offset in an upward helical trend.

After repeating the above operation to obtain the offset contours of all layers, the generation of the helical path starts. As shown in Figure 7, the current layer contour $P_{i}$ is derived from the horizontal offset of $Q_{i}$, which completes the contour offset stage in the hybrid path, and the next step needs to determine the longitudinal displacement of each point on the path according to the next layer contour $P_{i+1}$ to complete the helical connection stage in the hybrid path.

Because the contour is composed of a set of ordered points, every two of these ordered points can form a line segment. We take the $j^{th}$ point $P_{i,j}$ in layer $i^{th}$ as an example and calculate its distance from each point on the next layer of the contour and find the nearest point $P_{i+1,j}$. Let point $P_{i+1,j}$ and point $P_{i+1,j-1}$ form the line segment point $S_{j-1}$, and let point $P_{i+1,j}$ and point $P_{i+1,j+1}$ form the line segment point $S_{j}$. Then, we use point P_{i,j} to project the two line segments to obtain the projection points of both of them. The closest of these two points to the point $P_{i,j}$ replaces $P_{i+1,j}$ as the closest point on the next layer contour. As a result, the vector formed by point $P_{i,j}$ and point $C_{i,j}$ is the moving direction of point $P_{i,j}$.

The distance moved by point $P_{i,j}$ is not the distance between it and point $C_{i,j}$, but it needs to be additionally multiplied by the ratio of the cumulative length $L_{i,j}$ of the location where it is located to the total length $L_{i}$ of the contour. If the current point is the mth point of the contour, the cumulative length $L_{i,m}$ is derived according to Equation (5). If the current layer contour has n points, the total length $L_{i}$ is derived according to Equation (6). Finally, the helical path point $P_{helix}$ is solved according to Equation (7). The remaining points on the current layer contour are determined in the same was as point $P_{i,j}$ to obtain the corresponding points on the helical path, and for the rest of the layer, contours are treated in the same way as layer $P_{i}$.

(5) $$L_{i,m}=\sum_{j=0}^{m-1}\sqrt{{\left(x_{P_{i,j+1}}-x_{P_{i,j}}\right)}^{2}+{\left(y_{P_{i,j+1}}-y_{P_{i,j}}\right)}^{2}+{\left(z_{P_{i,j+1}}-z_{P_{i,j}}\right)}^{2}}$$

(6) $$L_{i}=\sum_{j=0}^{n-1}\sqrt{{\left(x_{P_{i,j+1}}-x_{P_{i,j}}\right)}^{2}+{\left(y_{P_{i,j+1}}-y_{P_{i,j}}\right)}^{2}+{\left(z_{P_{i,j+1}}-z_{P_{i,j}}\right)}^{2}}$$

(7) $$P_{helix}=P_{i,j}+\frac{L_{i,m}}{L_{i}}\vec{P_{i,j}C_{i,j}}$$

### 2.3. Tool Orientation Generation

When manufacturing an unsupported overhanging structure, it is necessary to match each point on the path with a reasonable tool orientation. To achieve a reasonable standard, the tool orientation must fit the surface of the part model where it is located. Since the surface curvature change is fitted to a triangular facet, the tool orientation must be generated concerning the normal vector of the triangular facet where the point is located. Before the tool orientation is formally generated, in the same way as path planning, the tool orientation for each point on the offset contour needs to be obtained as an initial value.

The purpose of the contours in the hybrid path needing to be offset is to account for the width of the melt path, but the corresponding tool orientations at each position are still adapted to the original surface of the model, not the offset surface. Therefore, the tool orientations on the offset contours are derived from the original contours. In the slicing phase, when each line is intersected with a plane, the two neighboring triangular facets to which it belongs are directly marked. After obtaining the normal vectors of the neighboring triangular facets, the corresponding tool orientation vectors $N_{i,j}$ for each deposition position on the original contour are calculated according to Equation (8).

(8) $$N_{i,j}=\frac{\left(n_{i,j-1}+n_{i,j}\right)\times \left(\vec{P_{i,j}P_{i,j+1}}+\vec{P_{i,j-1}P_{i,j}}\right)}{2}$$

where $n_{i,j-1}$ and $n_{i,j}$ are both triangular facet normal vectors associated with point $P_{i,j}$, and a cross-product operation between their average and the contour tangent vector of the point’s location can compute the tool orientation that fits the model surface.

Then, the tool orientation on the original contour is then delivered to the offset contour. Although the two contours have the same orientation, the number and order of points on them no longer correspond one to one. Therefore, it is necessary to re-search the position of the point on the offset contour on the original contour and assign the tool orientation at that position to the offset contour. Taking point $P_{i,j}$ on the offset contour as an example, when generating the helical path in the previous section, it is searching for its nearest point on the next layer of the contour, and similarly finding the nearest point $C_{i,j}^{\prime }$ on the original contour. This is because there are two possibilities for point $C_{i,j}^{\prime }$ to be either on the endpoints of the contour segment or between the two ends of the contour segment. If it is the former, only the tool orientation on the endpoints needs to be delivered to point $P_{i,j}$. If it is the latter, the average sum of the tool orientations at the ends of the line segment needs to be delivered to the point $P_{i,j}$.

**Figure 8:**
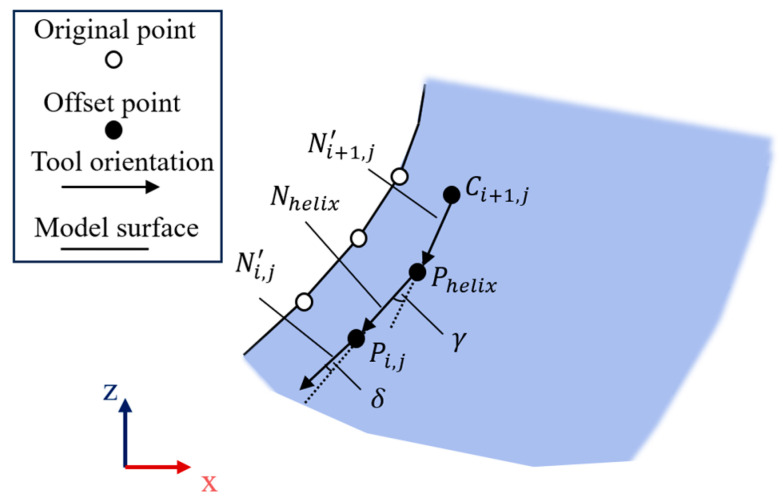
Computation of multi-axis tool orientations. Match vectors are used as tool orientations for each point on the hybrid path to apply the multi-axis strategy.

After obtaining the tool orientations $N^{\prime }$ on the offset contours of each layer, as shown in Figure 8, we emulate the principle of generating helical styles in a hybrid path and start matching the tool orientations for each path point.

Given the tool orientation $N_{i,j}^{\prime }$ of the current layer and the tool orientation $N_{i+1,j}^{\prime }$ of the next layer, we can solve for the tool orientation $N_{helix}$ of the helical path. We then obtain the angle γ between $N_{i,j}^{\prime }$ and $N_{i+1,j}^{\prime }$ according to Equation (9), and multiply it by the ratio of the cumulative length $L_{i,m}$ to the total length $L_{i}$ to obtain the angle of rotation δ. As shown in Equation (10), we rely on quaternion to complete the rotation, with q being the unit quaternion and A being the vector perpendicular to the vector $N_{i,j}^{\prime }$ of the unit vector, and δ is the angle at which $N_{i,j}^{\prime }$ rotates around A. The rotation is accomplished by the use of a quadratic, and the vector $N_{helix}$ satisfies Equation (11), which is obtained and then matched to the path point $P_{helix}$. This part of the method regarding the rotation of quaternions is referred to in [20] and will not be repeated here.

(9) $$\gamma =acrcos\left(N_{i,j}^{\prime }\cdot N_{i+1,j}^{\prime }\right)$$

(10) $$q=\cos\left(\frac{\delta }{2}\right)+Asin\left(\frac{\delta }{2}\right)$$

(11) $$N_{helix}=qN_{helix}q^{-1}$$

Tool orientation has two methods of expression, in addition to the vectorial way in the workpiece coordinate system and the way it is decomposed into the angle of rotation of the equipment components in practical applications. Due to the barriers between digital and physical space, additional consideration needs to be given to the motion characteristics of multi-axis equipment. Taking a multi-axis device with two rotational axes as an example, although the trend of tool orientation will adapt to the curvature of the surface of the part model, there may be a problem of sudden change in the rotational angle when decomposed into the angle of the two rotational axes. 

**Figure 9:**
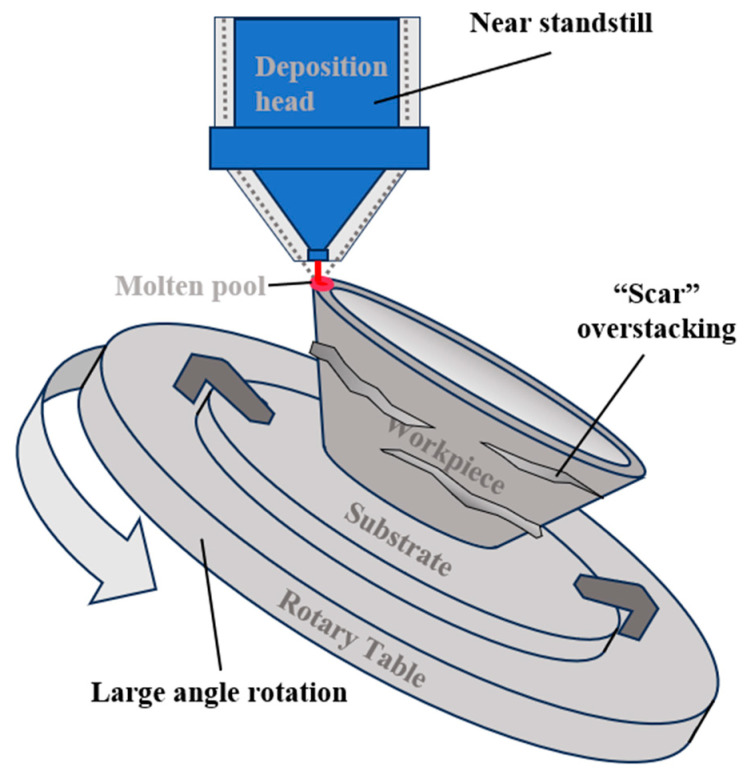
Formation of “scar” defects. The moving axis waits for the rotary axis to complete a large angular movement that will continue to melt more metal material.

As shown in Figure 9, the problems associated with abrupt changes in the angle of rotation are illustrated. In multi-axis linkage technology, the ability of the laser deposition direction and position to conform to program instructions is the result of the collaborative work of multiple axes. The planned deposition path has a close distance between the coordinates of neighboring positions, but the tool orientations at neighboring positions may vary widely when decomposed into angular combinations, which for rotary base equipment suitable for the LMD process is specifically manifested in the motion of the rotary table. If the moving axes arrive at the specified position at their original speed, the rotary axes may not have completed their motion due to the upper speed limit that exists for each axis. As a result, the laser deposition head will move at an almost stagnant speed for a very short displacement distance until the rotary table is rotated to the specified angle. The laser does not turn off as a result, causing the melt pool to melt more metal powder at that location and thus creating an overstack. On overhanging thin-walled parts, this manifests itself in the form of “scar” defects on the part surface that resemble the laser’s trajectory.

Therefore, the optimization and constraints will be performed using the kinematic forward and inverse solutions [21]. The rotation angle data obtained from the forward solution are extracted separately to simplify the problem model, and the trend of the rotation angle change is expressed using a folded line. The smoothed angles are obtained by an inverse solution to obtain the updated tool orientation and compare it with the original tool orientation to constrain the degree of smoothing.

**Figure 10:**
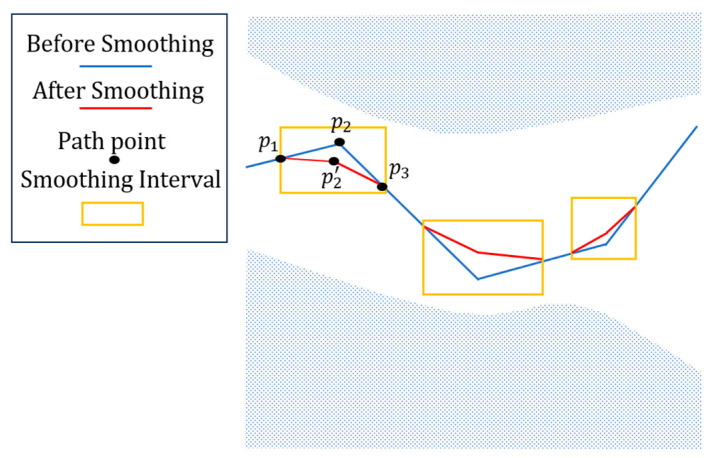
Smooth effect. The two colored lines represent before and after smoothing, optimized by two changes in rotation angle to achieve tool orientation.

As shown in Figure 10, areas with large variations are detected and eliminated based on the slope of the fold line. The shaded area is the part where the fold line is prohibited, the yellow box is the interval to be smoothed, the blue line represents the initial value before smoothing, and the red line is the effect after smoothing. Point $p_{1}$ and point $p_{2}$ form a line segment, as do point $p_{2}$ and point $p_{3}$, which have a slope ratio above the given threshold. Therefore, averaging is performed based on the angular difference from $p_{1}$ to $p_{3}$, and the intermediate point $p_{2}$ is updated to $p_{2}^{\prime }$, thereby completing the smoothing process.

## 3. Result

### 3.1. Computer Output NC Program

**Figure 11:**
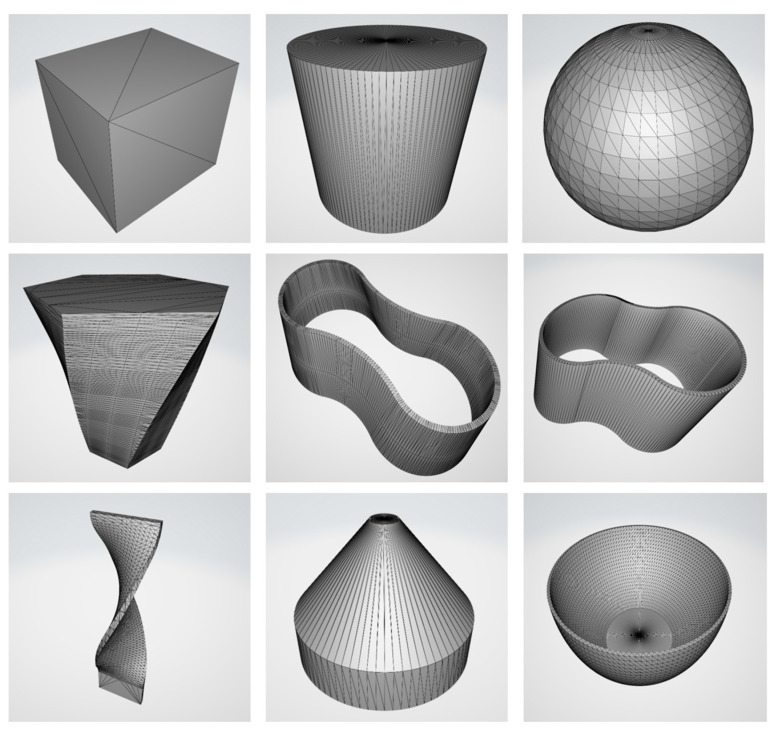
Part models in STL format. They have different geometric features and are used to validate the path generation.

To evaluate the path generation method proposed in this paper, it was run on a laptop configured with i9-12900H 2.50 GHz CPU and 16.0 GB of RAM. Visual Studio 2022 was used as a development environment using the Windows 11 (64-bit) operating system. The CAD/CAM content involved in the whole work is developed in C# code language for intelligent and automated output of NC programs to the machine tools. We input the part model in STL format and visualized the output path data. Models with different geometrical features were selected for testing and their STL models are shown in Figure 11, and the triangle mesh covering the model was used to fit its surface curvature variations.

**Figure 12:**
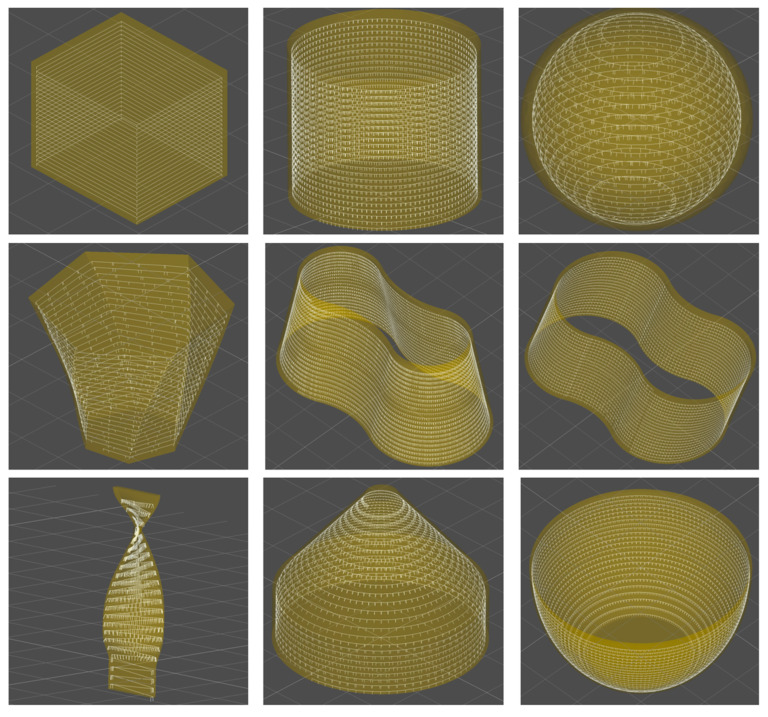
Generation of multi-axis hybrid paths. Display offset and helical effects and attach multi-axis tool orientations to them.

Then, different strategy parameters are set to generate the deposition path. As shown in Figure 12, the yellow geometry is the model of the part, the white helix is the path, and the vertical lines on the helix are the tool orientation vectors. The white helical upward path does not follow the outer surface of the model itself, but is a given distance from the outer wall, which is the effect of the contour offset strategy. The white vertical lines on the path fit both the curvature variations of the model’s surface and are perpendicular to the boundary of the deposition layer, which ensures a reasonable buildup of material at that location.

**Figure 13:**
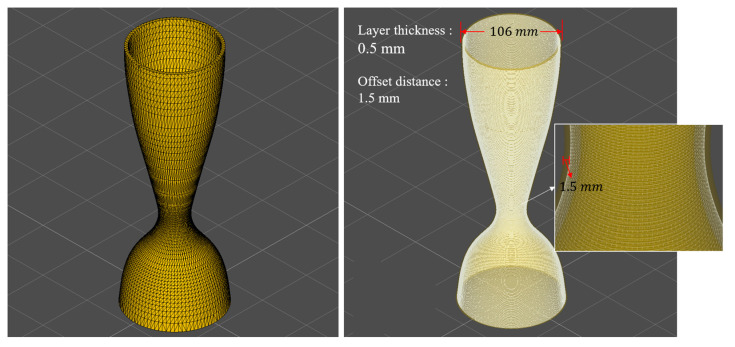
Generation of paths for the rocket thruster. Setup parameters commonly used by the process for practical application.

After confirming that the helical paths can be generated correctly, the planning of the process flow for the actual application begins. As shown in Figure 13, the hybrid path was generated based on the STL model of the rocket thruster. The parameters of the path strategy include a layer thickness of 0.5 mm and a spot diameter of 3 mm. Since the laser scanning path point is at the center of the spot, the strategy automatically determines the offset distance to be 1.5 mm after the process parameters of spot diameter are given.

**Figure 14:**
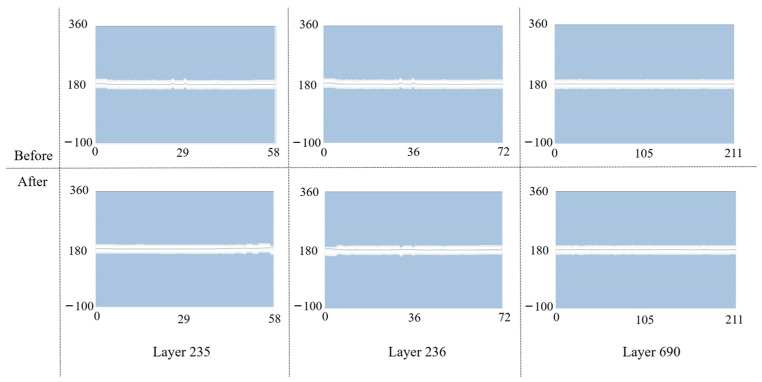
Smoothing of the A-axis angle. Examples are given of smoothing effects in certain regions, which are characterized by the need to deal with only small fluctuations.

The path data obtained in the work coordinate system also require post-processing before it can be recognized by the machine. Although the coordinate points on the path do not require additional transformations due to the RTCP, the tool orientation needs to be decomposed from the vector state to the degrees of the two rotation angles. Therefore, the vector orientation on the path is orthogonalized to obtain the angles of the A-axis and C-axis. Taking layers 235, 236, and 690 as an example, as shown in Figure 14, the vertical axis is the range of rotation angles of the rotary table around the A-axis, the horizontal axis is the total number of path points in this layer, and the broken line represents the trend of the A-axis angle. Regarding the background color, white represents that it is a safe area, blue represents the laser leakage area, and gray represents the transition area. The results show that the small fluctuations present in the A-axis angle change are smoothed out and remain in the safe region.

**Figure 15:**
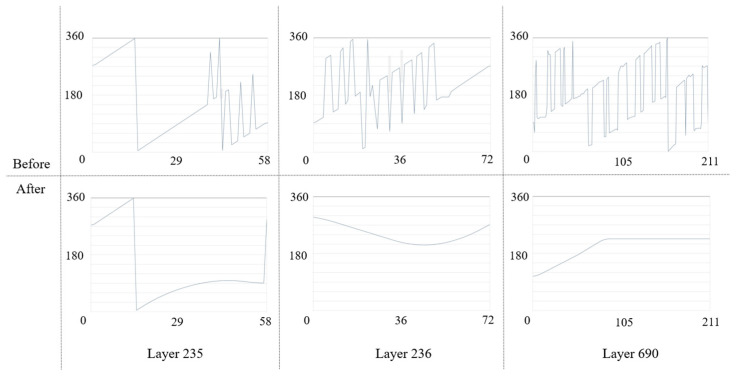
Smoothing of the angle of the C-axis. Example of a smoothing effect for a certain number of regions, which is characterized by handling sharp corners and large fluctuations.

Because tool orientation consists of two rotation angles linked together, optimizing the A-axis angle is automatically completed with a smoothing operation for the C-axis angle. As shown in Figure 15, the vertical axis is the angle range of rotating the rotary table around the C-axis, and the horizontal axis is the number of all the path points of the layer. The results show that both small and large fluctuations in the C-axis angle change are smoothed and the $360^{{}^{\circ}}$ to $0^{{}^{\circ}}$ or $0^{{}^{\circ}}$ to $360^{{}^{\circ}}$ cases will be retained because the machine tool usually moves in the direction of the smallest rotation angle.

**Figure 16:**
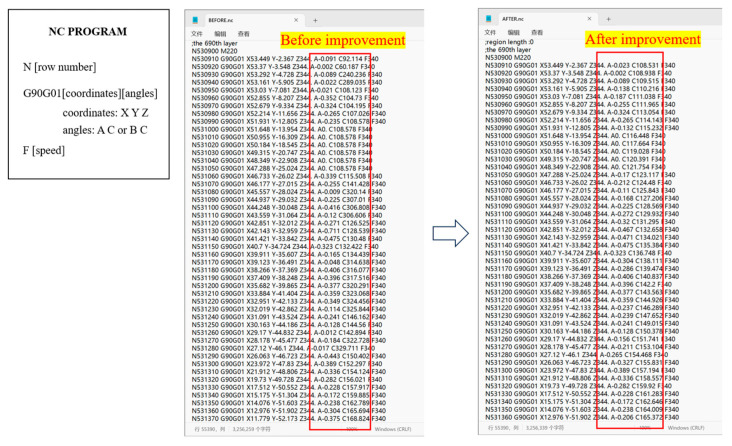
Output of NC program. This file enables the path to be recognized by the equipment so that improvements and updates of angles are reflected by changes in the data in the file.

The angles of the A-axis and C-axis are applied to the NC program after the update. As an example, the $690^{th}$ layer is shown in Figure 16, which shows two program instruction files before and after the optimization. It is possible to observe that the values of the angles of the two rotary axes have been changed, replacing the set of values with a larger variation with another set of values that are smoother.

**Figure 17:**
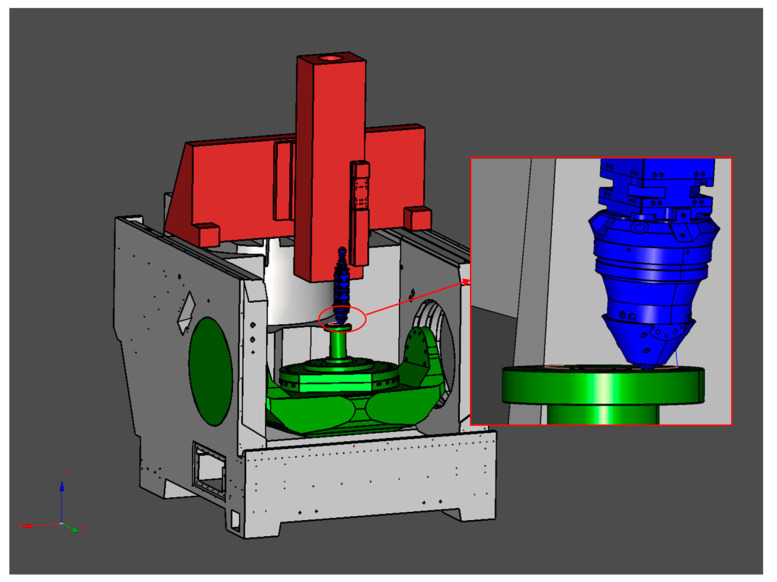
Machine tool simulation. Checking paths through the state of the machine component linkage, in order to detect problems in advance.

The path is then confirmed in a simulation environment also developed in C#. As shown in Figure 17, the model of the machine tool that will be used later is imported, and the movement of the deposition head according to the path is observed and analyzed to identify and solve problems before they are applied. Simulation is used to maximize time and energy cost savings.

### 3.2. Machine Applied to the NC Program

**Figure 18:**
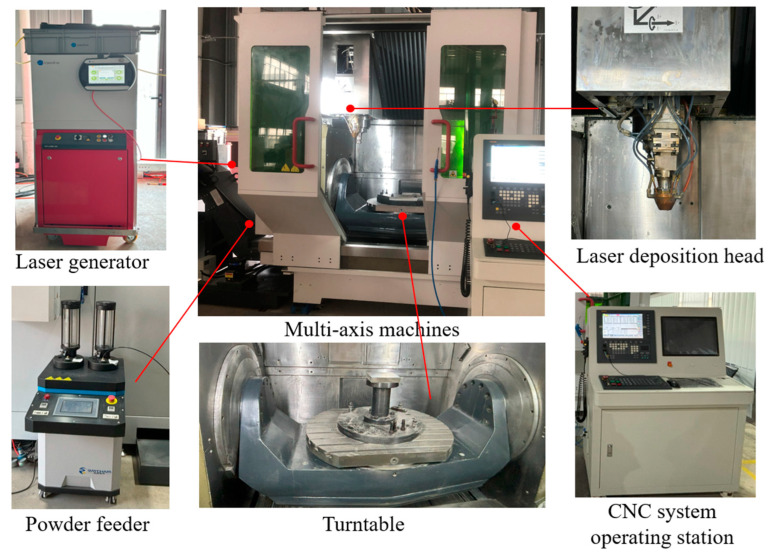
Selected multi-axis equipment. A 5-axis machine with LMD process and zoomed in to show major details.

The machine chosen was a coaxial powder feeder, as shown in Figure 18, which consists of five main important components. The laser equipment cooperates with the powder-feeding equipment in order to supply the deposition head with high-energy laser and metal powder material. The metal traces left along the path are able to accumulate to form parts on the substrate surface of the turntable. The computer-generated path file is transferred to the numerical control system, which allows the laser deposition head and the rotary table to move according to the prescribed program instructions.

This section of the experiment is designed to address three main aspects, which are described in descending order of importance. First, we aim to verify that the path generated by the proposed algorithm is capable of performing the task of forming overhanging thin-walled structures, which is also the main objective. Second, we aim to verify the feasibility of the contour strategy introduced into the helix strategy, which is used to differentiate it from previous conventional methods that do not take the offset into account, taking into account the fact that the width of the melt path in the LMD process cannot be ignored. Thirdly, the validation of the rotational angle smoothing, which takes into account the linkage of the multi-axis equipment movements, is used to improve the “scar” type of overstacking of the paths in practical applications. The achievement of these three aspects is demonstrated by the successful printing of a complete thin-walled overhanging metal part with insignificant “scar” on the surface of the part and a few macroscopically visible imperfections.

**Figure 19:**
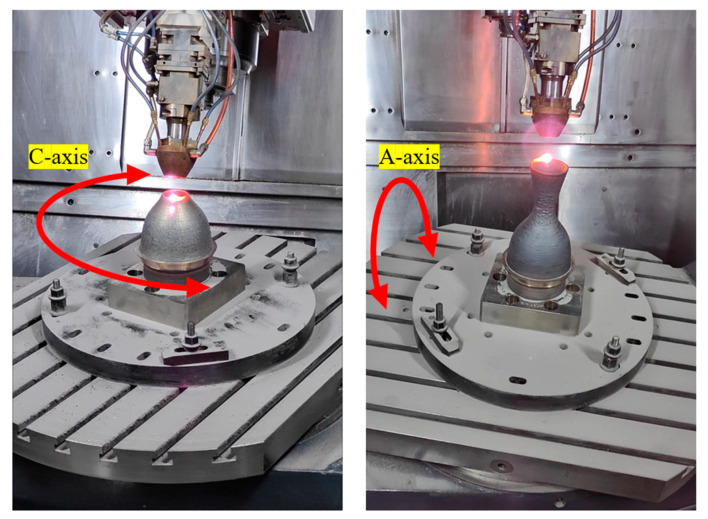
The procedure of forming a part. The laser and powder form a molten pool, which enables the metal material to melt and build up in a specified manner.

In order to verify the expected results outlined above, two paths are designed to be applied to the actual device. The target part is a rocket thruster, which is a thin-walled hollow structure with overhanging and curved surface features. The difference between the two paths lies in two points. The first one is close to the previous method, which directly outputs the helical path according to the model boundary without optimizing the tool orientation. The second is the method proposed in this paper, where the helical path is pre-offset according to the spot radius parameter taking into account the melt path width, and the output tool orientation is optimized by rotational angle smoothing. The processing parameters are based on conventional experience, whereby the laser power is set to 700 w and the scanning speed is set to 600 mm/min. Under these same process parameters, two paths are used to manufacture the same part. As shown in Figure 19, the LMD process is used in the fabrication of rocket thrusters. It is not advisable to change the orientation of the laser and the powder, and to ensure that the melt pool is formed in the correct position, a rotating base is used to change the relative position of the deposited surfaces, thus having the effect of changing the tool orientations. The two additional rotary axes are the A-axis and the C-axis, and the rotary table drives the substrate and workpiece around them in a rotary motion according to NC program commands.

**Figure 20:**
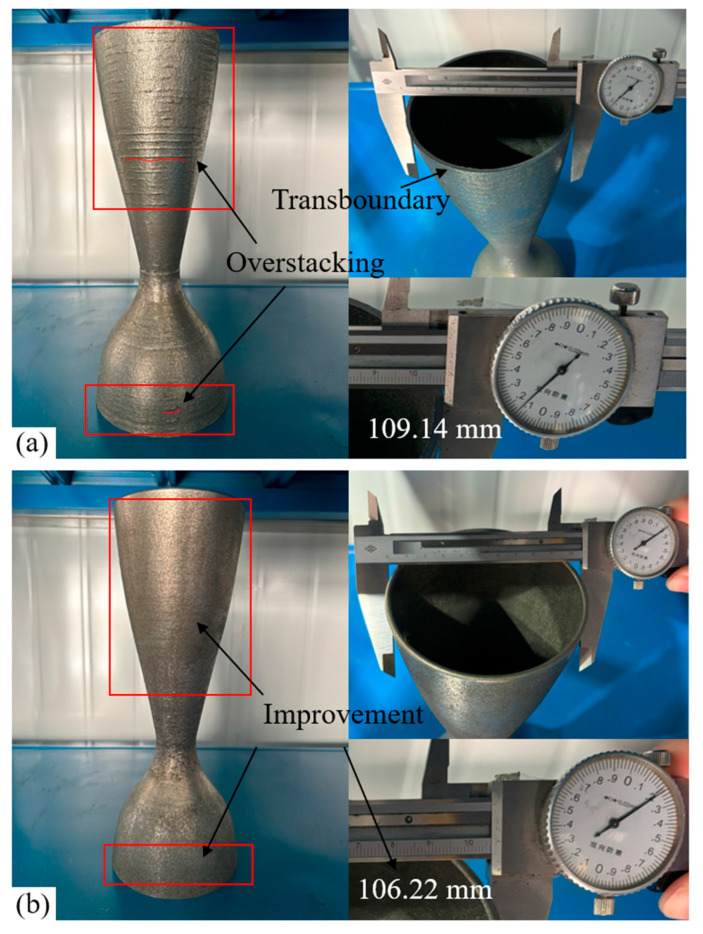
Manufactured rocket thruster. A multi-axis machine reads two different paths to form the part to demonstrate the feasibility and effectiveness of the method. (**a**) The result of the application of the first path. (**b**) The result of the application of the second path (proposed method).

The results of the two path data applications are shown in Figure 20, in which Figure 20a shows the results of the first comparison path and Figure 20b shows the results of the second path generated by the proposed method. In the red box area, the surface of the part printed with the first method has more “scar” type defects, which is an overstacking problem and seriously affects the surface quality. The part printed by the second path has fewer “scar” defects on the surface, and there is almost no problem with over buildup. Measurements of the two parts show that the top layer of the first method has a diameter of 109.14 mm, which is 3.14 mm more than the pre-determined contour width of 106 mm in the CAD model. The top layer of the second one measured 106.22 mm in diameter. The experimental results show that the proposed method is able to successfully fabricate overhanging thin-walled structures and that it has a macro-visible effect on the “scar” defects on the surface of the part. In addition, it was demonstrated that deposition at the indented channel radius width resulted in parts formed by the LMD process that were closer to the desired dimensions than the previous method of directly following the model boundaries.

## 4. Conclusions

In this study, a multi-axis hybrid path generation method is provided for the LMD process to form unsupported overhanging thin-walled structures. The proposed method aims to generate paths through computerized geometric algorithms, including slicing, path planning, tool orientation matching, and post-processing, with each stage solving different geometric problems to achieve a divide-and-conquer effect. In the slicing stage, not only the contour of each layer of the part model is obtained by layering but also the adjacency between the triangular facets is utilized to reduce the redundant intersection computation in planes and meshes. In the path-planning stage, a new spatial hybrid path is formed by combining the contouring and helix strategies, taking into account the continuity of the path and the geometric reduction in the boundary in order to avoid the frequent starting and stopping of the laser during the forming process of the thin-walled structure, and the cross-boundary of deposition in the contour area caused by the width of the melt path. When introducing the multi-axis strategy, the path is matched with tool orientation vectors that fit the curvature of the model surface to ensure it is competent for overhanging structures, and the motion angle of the rotary axes is smoothed to improve the “scar” overstacking phenomenon on the thin-walled surface, taking into account the characteristics of the multi-axis linkage motion.

All the theoretical logic was first confirmed by modeling different geometrical features to confirm its feasibility and then applied to fabricating metal parts in the LMD process to verify its validity. In the experimental phase, the target part chosen was a rocket thruster, which is a thin-walled structure with overhanging and curved features. The paths generated by the multi-axis machine following the proposed methodology allowed the part to be successfully shaped from a digital model into a physical entity with contour boundary dimensions close to those expected from the CAD model, and the part surface eliminated “scar” overstacking to maintain a clean appearance in terms of macroscopic visibility. In the future, in order to demonstrate the potential of the proposed methodology, the strategy will continue to be refined for the fabrication of more complex thin-walled parts.

## Data Availability

The original contributions presented in the study are included in the article, further inquiries can be directed to the corresponding author.
